# Veterinary and technical optimization of the fetal sheep model of congenital diaphragmatic hernia: implications for translational pediatric surgery

**DOI:** 10.3389/fsurg.2025.1711870

**Published:** 2025-12-03

**Authors:** T. Connor McCorkell, Daniela Espinosa Seoane, Elke Zani-Ruttenstock, Fabian Doktor, Rebeca Figueira, Melissa Sinclair, Alex zur Linden, Marta Horna, Lucciana Recchi, Alice Defarges, Lina Antounians, Andreana Bütter, Augusto Zani, Judith Koenig

**Affiliations:** 1 Department of Clinical Studies, Ontario Veterinary College, The University of Guelph, Guelph, ON, Canada; 2 Division of Pediatric Surgery, Children’s Hospital, LHSC, Schulich School of Medicine & Dentistry, Western University, London, ON, Canada; 3 Division of Pediatric Surgery, Hospital for Sick Children, The University of Toronto, Toronto, ON, Canada

**Keywords:** congenital diaphragmatic hernia, pulmonary hypoplasia, sheep, lamb, fetal surgery, pediatric surgery, veterinary medicine, translational animal models

## Abstract

Congenital diaphragmatic hernia (CDH) is a life-threatening developmental anomaly where abdominal organs herniate into the thoracic cavity, impairing fetal lung growth and subsequent postnatal lung function. Despite advances in treatment, the morbidity and mortality of CDH remain significant. Currently, the most well-established fetal intervention is fetoscopic endoluminal tracheal occlusion (FETO), which promotes lung expansion and development by temporarily blocking the egress of lung fluid. However, treatment outcomes remain variable, which underscores the need for robust animal models to investigate novel therapies. The fetal sheep model is particularly valuable due to physiological similarities to human infants in lung development and anatomy. However, its successful implementation requires substantial veterinary and surgical expertise. In this paper, we outline the surgical protocol, refinements, and perioperative challenges in establishing a fetal sheep model of CDH to test a novel therapy. A diaphragmatic defect was surgically created via fetal thoracotomy at 80 days of gestation using a maternal caudal ventral midline laparotomy. Fetal tracheal occlusion with treatment administration was performed via a maternal left flank laparotomy at 108 days, followed by euthanasia then delivery at 136 days. Initial surgeries experienced complications such as maternal incisional dehiscence and herniation. These were mitigated through changes in surgical approach, closure techniques, and enhanced postoperative care. Veterinary oversight was critical in optimizing maternal well-being, minimizing stress, and improving recovery outcomes. This refined model provides a reproducible, welfare-centred approach integrating essential veterinary contributions to support translational pediatric surgery research in CDH.

## 1 Introduction

Congenital diaphragmatic hernia (CDH) remains a life-threatening condition wherein a defect in the diaphragm enables abdominal viscera to herniate into the thoracic cavity, preventing normal lung development. Despite significant advancements in neonatal care and surgical management, CDH remains associated with a mortality rate of approximately 20% (1), primarily due to respiratory failure from severe pulmonary hypoplasia and pulmonary hypertension (2).

Fetoscopic tracheal occlusion (FETO) has emerged as a promising prenatal therapy, whereby a temporary balloon is placed in the fetal trachea to prevent the egress of lung fluid and to stimulate lung expansion (3). However, FETO alone does not fully rescue lung maturation and vascularization, and it has limited effects on preventing postnatal pulmonary hypertension (3). To address these limitations, a range of therapeutic strategies are under development, spanning regenerative medicine to pharmacologic interventions (4), which may promote lung vascularization and functional maturation.

Animal models have played an important role in better understanding the pathophysiology of CDH and in developing and refining prenatal interventions. Rodent models are economical and have a short gestation period, enabling a greater sample size. However, rodent models are typically based either on genetic models of CDH or on administration of the teratogen nitrofen to induce the diaphragmatic defect, the latter of which can lead to off-target effects such as congenital heart defects (5, 6), also seen in human CDH (7). However, nitrofen acts through retinoic acid disruption, a pathway not clearly implicated in human CDH (8), thus its translational relevance may be limited. Rodents are also physiologically distinct from human neonates, particularly in lung development and size (9). Both rabbits and sheep are established surgical models of CDH since they each undergo lung development more closely resembling that of humans compared to rodents (10, 11). The rabbit CDH model is lower cost, with shorter gestation periods and larger litter sizes than sheep (12). However, sheep are considered a more clinically robust translatable animal model for human CDH infants due to their larger fetal size, longer gestation period, and greater similarity for lung anatomy and physiology than other animal models (13). These characteristics make fetal sheep particularly well suited for surgical interventions, diagnostic imaging, and longitudinal assessments that parallel human approaches.

First described in 1967 by de Lorimier et al. (14) the surgical fetal sheep model of CDH continues to serve as a valuable model for studying CDH pathophysiology and evaluating prenatal interventions (12, 13, 15). However, this model presents significant technical and perioperative challenges, including animal welfare considerations, postsurgical complications, and fetal loss. In many previous reports, maternal perioperative management, such as maternal surgical approach, postoperative analgesia, animal welfare and recovery monitoring, has been described only briefly or with limited detail (14, 16–18).

However, maternal stress and postoperative comfort can influence pregnancy maintenance and fetal well-being (19, 20). In previous studies, fetal loss has been reported to vary significantly, with mortality rates ranging from 10% to 50% (17, 18, 21–23), largely due to preterm labor (18, 21), abortion (17, 18, 21, 23, 24), and fetal demise following defect creation and/or therapeutic intervention (17, 18, 21–23, 25). In addition, methodology differences between research groups, including incision approach, uterine handling, perioperative antibiotics, and pain management, can make study replication challenging. Given this, successfully implementing this surgical model requires a multi-disciplinary approach among scientists, pediatric surgeons, and veterinary specialists. In this paper, we describe a refined fetal sheep model of CDH, with particular emphasis on the veterinary aspects of the model, including anesthesia and analgesia, surgical techniques, and maternal care. Veterinary expertise is integral to the success of this model, helping to ensure animal welfare, improve consistency, and enhance its translational relevance to humans.

## 2 Materials and methods

### 2.1 Animal ethics

All animal procedures were carried out in strict accordance with the guidelines of the Canadian Council of Animal Care (CCAC) and institutional protocols for the care and use of animals. These procedures were approved by the Animal Care Committees of the University of Toronto (Animal utilization protocol; AUP # 655371) and the University of Guelph (AUP # 5059).

### 2.2 Animals and housing

**Figure 1:**
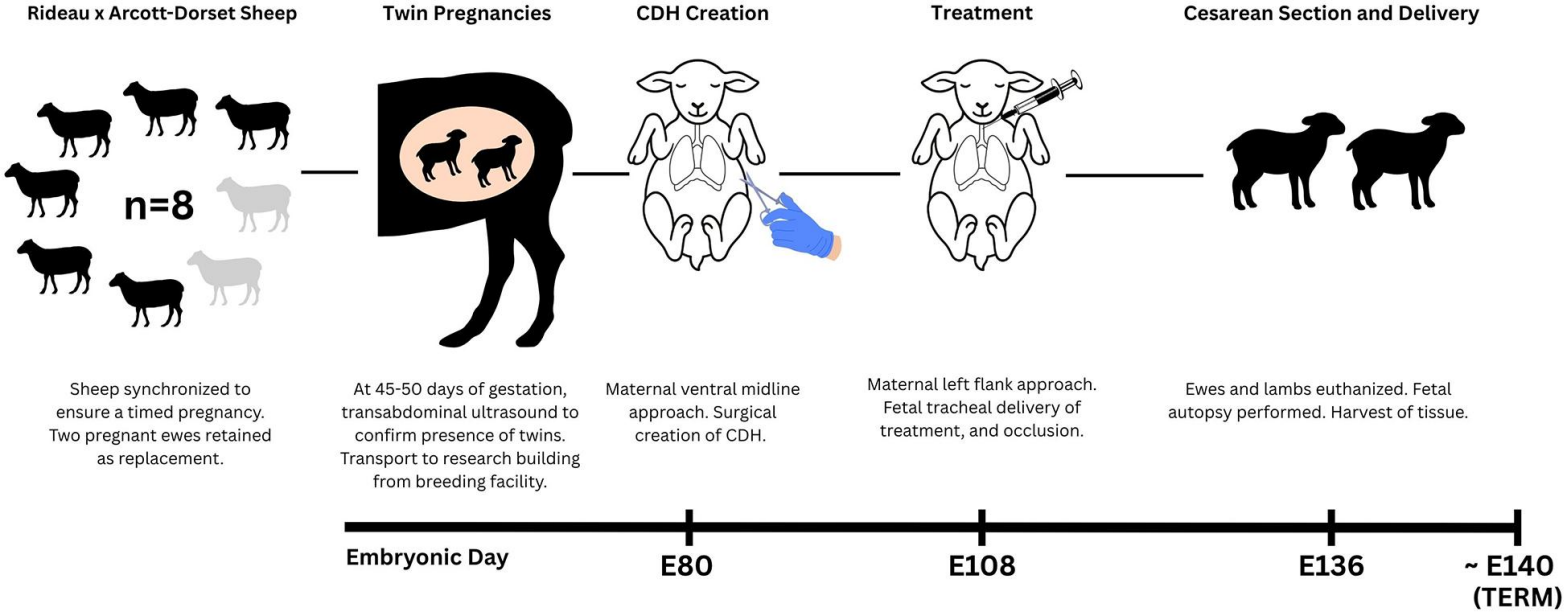
Study timeline showing key events in the surgical model of CDH.

Eight female Rideau Arcott-Dorset cross sheep from the university-owned pathogen-free flock of the Ontario Sheep Research Centre (Ponsonby, ON) weighing a mean body weight (BW) of 86 kg (range: 78–98 kg) were used. The sheep were synchronized to ensure a timed pregnancy, with a total of eight ewes and sixteen lambs; two of which were retained as backups in the event of fetal or maternal loss, yielding an experimental sample size of six ewes and twelve lambs. At 45–50 days of gestation, twin pregnancies were confirmed via transabdominal ultrasonography. All ewes were assessed and deemed clinically healthy by research station technicians, and then they were sheared before transportation to the Central Animal Facility (University of Guelph, Guelph, ON) 7–9 days prior to surgery to allow for adequate acclimatization (Figure 1). On arrival, ewes were weighed, assigned a body condition score (BCS), and received a physical examination by the veterinary team. Ewes were housed in groups of 2–4 per pen in a climate-controlled room with a 12-hour light/dark lighting cycle. Pens were bedded with wood shavings and cleaned twice daily. Ewes had *ad libitum* access to timothy-alfalfa hay, fresh water, and a salt lick. Jolly balls, daytime background music, slow feeding hay nets, and daily interaction with staff were some of the methods of environmental enrichment provided.

**Figure 2:**
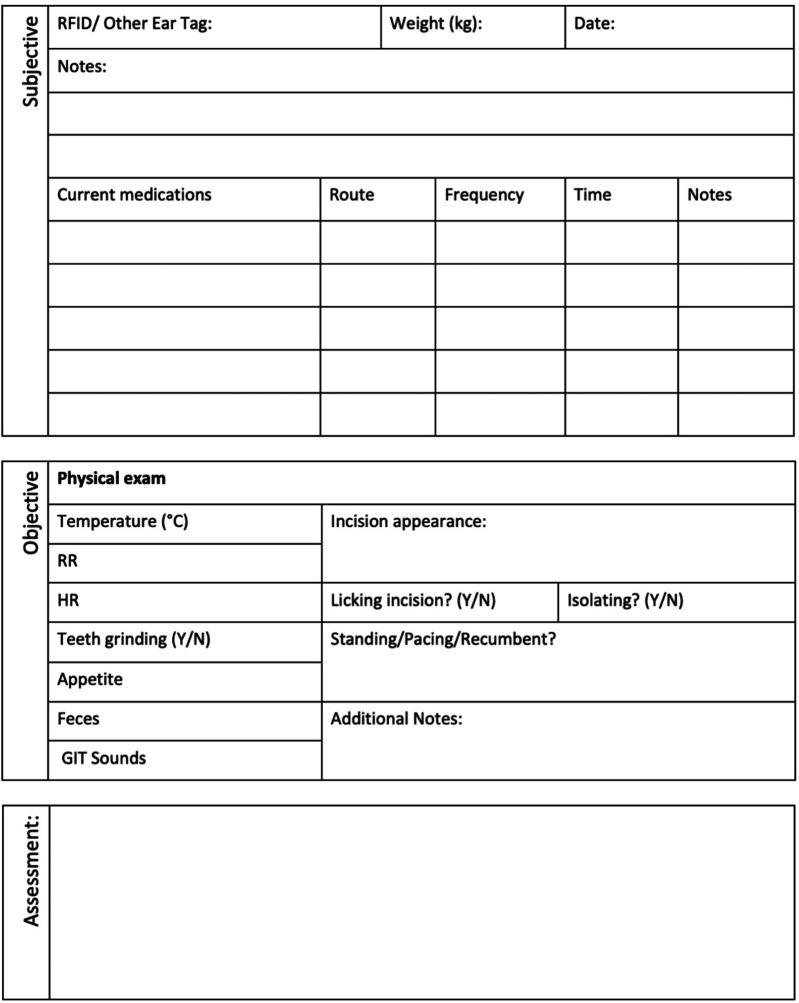
Standardized SOAP (subjective, objective, assessment, plan) animal assessment sheet used for post-surgical monitoring.

**Figure 3:**
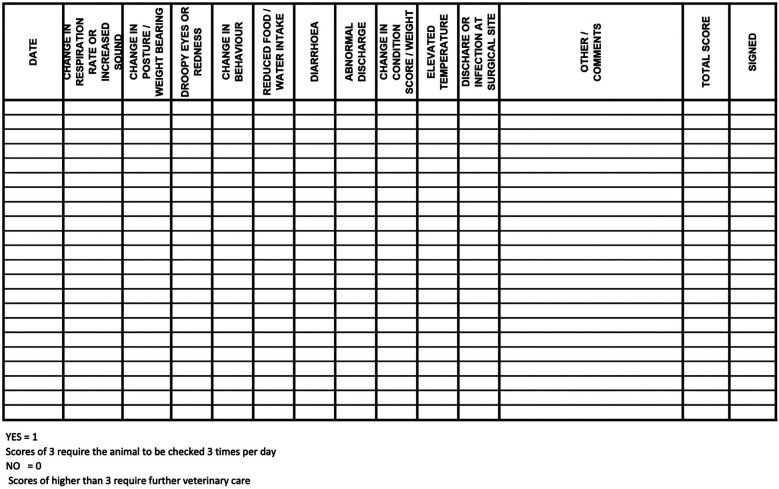
Postoperative day-to-day assessment log used for monitoring ewes following abdominal surgery. Each criterion was scored as either 0 (normal) or 1 (abnormal), with cumulative scores guiding monitoring frequency. Ewes with scores of 3 were assessed three times daily, while scores higher than 3 triggered immediate veterinary evaluation.

Baseline assessments were conducted in the days leading up to surgery by the veterinary team to establish preoperative clinical status and to acclimate the ewes to routine handling. These assessments included heart rate (HR), respiratory rate (RR), ruminal contraction rate, rectal temperature, mucous membrane colour, capillary refill time, feed and water intake, fecal consistency, and urination if observed. Oat grain was offered during these assessments to encourage cooperation and establish positive human-ewe interactions. Assessments followed established behavioural and physical welfare indicators for sheep (26, 27), with particular attention to signs of post-operative pain or discomfort such as teeth grinding, low ear and head position, prolonged recumbency, reluctance to rise, pawing, incision licking, or social withdrawal. All observations were documented twice daily using a standardized SOAP (Subjective, Objective, Assessment, Plan) format and a day-to-day assessment point system post-surgery, both of which were maintained in each ewe's medical record (Figures 2, 3). The postoperative day-to-day assessment point system included a collection of clinical and behavioural parameters evaluations. Each criterion was scored as either 0 (normal) or 1 (abnormal), with cumulative scores guiding monitoring frequency.

### 2.3 Animal preparation and perioperative protocol

Twenty-four hours before surgery, ewes were moved to pre-operative holding pens adjacent to the surgical suite. This facility was directly connected to the main housing area, allowing for efficient and low-stress transfer. Ewes were moved in small groups (2–4 ewes) and guided loosely (i.e., without physical restraint) using grain as positive reinforcement. Throughout the perioperative period, ewes were housed in groups of 2–4 to avoid social isolation and promote their welfare. Feed was withheld for 24 h and water for 8 h in preparation for general anesthesia to prevent rumen distention and aspiration pneumonia from the reflux of rumen contents (28).

### 2.4 Analgesia and anesthesia protocol

#### 2.4.1 Sedation and pre-anesthesia

On the day of surgery, ewes were sedated with xylazine, (0.1 mg/kg, IM; Rompun, Elanco Ltd, Mississauga, ON) one hour before jugular catheterization. Skin over the jugular vein was clipped, and a topical local anesthetic cream (Maxilene; RGR Pharma Ltd, Canada) was applied for 10 min; alternatively, a bleb of lidocaine (20 mg/mL; Surgo Surgical Supply, Newmarket, ON) was administered subcutaneously (SC). A 16–18-gauge catheter (Insyte-W; Becton Dickinson Infusion Therapy Systems, UT, USA) was inserted and maintained in place with stay sutures. A light bandage was also placed over the catheter, allowing the ewes to remain loose within the holding pen.

#### 2.4.2 Anesthetic induction, maintenance and monitoring

Midazolam (0.02 mg/kg IV; Midazolam, 5 mg/mL, Sandoz) was administered for additional sedation prior to induction. Ewes were positioned in sternal recumbency and pre-oxygenated (4 L/min via facemask). General anesthesia was induced using a co-induction protocol of IV ketamine (2 mg/kg; Narketan, 100 mg/mL, Vetoquinol, Lavaltrie, QC) and propofol (1 mg/kg; Propofol 10 mg/mL; Pharmascience, Inc., Canada) to effect. Topical lidocaine was applied to the larynx, followed 30 s later by orotracheal intubation with a cuffed endotracheal tube (ET) (8.5–10 mm ID) under direct visualization with a laryngoscope. The ET cuff was rapidly inflated to protect the airway from aspirating secretions, then the endotracheal tube was connected to a rebreathing circuit (Universal F-Circuit; Dispomed, Canada) with an oxygen flow of 3.5–4 L/min. A manual breath with the anesthetic circuit re-breathing bag was administered to assess for an ET cuff seal at a peak inspiratory pressure (PIP) of 20 cmH_2_O prior to placing the ewe in dorsal recumbency. Maintenance with isoflurane (1%–3%; Aerrane, Baxter Corp, Mississauga, ON) delivered in oxygen (20–40 mLs/kg/min) was instituted with mechanical ventilation (Model 2002; Hallowell EMC, MA, USA). Target ventilator settings included a tidal volume of 15 mL/kg, respiratory rate of 6–8 breaths/min, and PIP of 15–20 cm H_2_O to maintain normocapnia (ETCO_2_ = 30–35 mmHg). Ewes were positioned with the occiput slightly elevated, and nose lowered as much as possible in dorsal recumbency to facilitate drainage of regurgitant fluid and minimize the aspiration risk. No neuromuscular blocking agents were used during anesthesia.

Monitoring included manual depth assessment of palpebral reflexes and visualization of esophageal contractions, HR, electrocardiography (ECG; lead II), oxygen saturation (SPO2), end-tidal carbon dioxide partial pressure (PE CO2) and RR using capnography (PM-9000 Express; Mindray Medical International Ltd, China), rectal temperature, and systolic arterial blood pressure (Doppler) measurements every 5 min.

#### 2.4.3 Surgical site preparation and analgesia

For each of the three surgeries, the surgical site was clipped (Wahl Professional Clippers, 10 and 40 blades) and prepared aseptically using 4% chlorhexidine soap and a subsequent final preparation consisting of 70% isopropyl alcohol and chlorhexidine pre-op solution (Chlorhexidine gluconate 0.5% w/v with 70% isopropyl alcohol).

**Figure 4:**
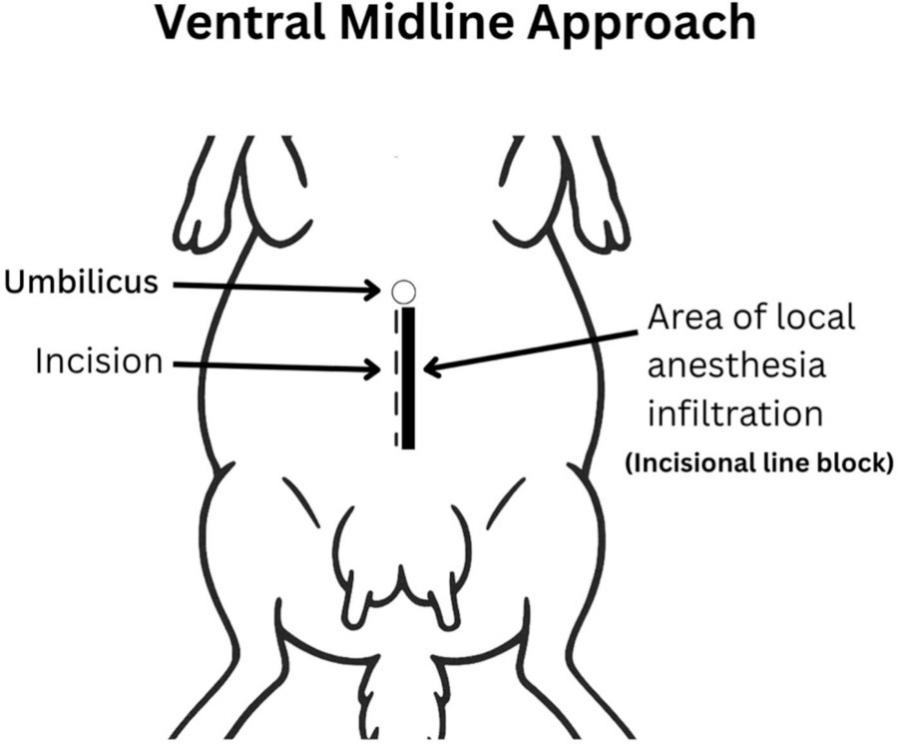
Ventral midline approach. The incision was made 2–3 cm caudal to the umbilicus along the linea alba and extended 15–20 cm caudally. Local anesthetic was infiltrated subcutaneously along the planned incision (line block) using bupivacaine (1 mg/kg SC; Pfizer, Kirkland QC).

**Figure 5:**
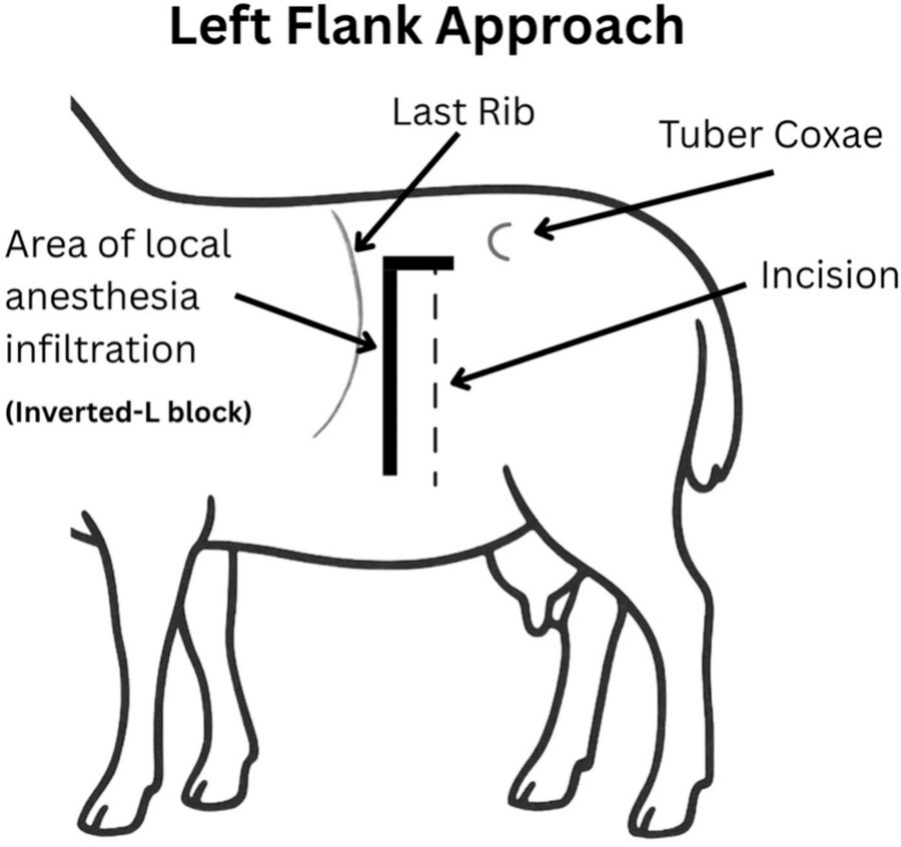
Left flank approach. A 15–25 cm vertical or slightly oblique skin incision was made, starting approximately 5–10 cm ventral to the lumbar transverse processes and centered within the paralumbar fossa. An inverted L block was performed for local anesthesia using bupivacaine (1 mg/kg SC; Pfizer, Kirkland QC).

Prior to surgical incision, ewes received hydromorphone (0.05 mg/kg, IV). A local incisional line block (ventral midline incision; Figure 4 or inverted-L block (left flank approach; Figure 5) with bupivacaine (1 mg/kg SC; Pfizer, Kirkland QC) was then administered. Intravenous lidocaine (2 mg/kg; Surgo Surgical Supply, Newmarket, ON) was administered before surgical incision to reduce the minimum alveolar concentration (MAC) in addition to Meloxicam (0.5 mg/kg; Metacam, 20 mg/mL; Boehringer Ingelheim Canada Ltd) and ceftiofur sodium (2.2 mg/kg IV; Excenel®, Zoetis Canada Inc., Kirkland, Québec, Canada).

Lactated Ringer's solution was administered intravenously at 3–5 mL/kg/h. Hypotension (systolic pressure <80 mmHg) was treated with dopamine (2–7 µg/kg/min) or dobutamine (0.5–5 µg/kg/min). Bradycardia (<45 bpm) was treated with glycopyrrolate (0.005 mg/kg IV). Normothermia (>36.5 °C) was maintained using heating pads and forced air warming devices.

#### 2.4.4 Recovery

After surgery, isoflurane was discontinued, and spontaneous ventilation resumed. Ewes were positioned in sternal recumbency and extubated once they exhibited signs of recovery, including head-lifting, chewing, eructation, and active swallowing. The endotracheal cuff was left inflated during extubation and inspected for signs of aspiration.

If ewes did not stand within 20 min postoperatively, atipamezole (0.005 mg/kg IV) was administered to reverse residual xylazine sedation. Hydromorphone (0.10 mg/kg; Sandoz, Canada Inc, Longueuil, QC) was administered as needed during the initial 2–3 h of recovery. Buprenorphine (0.005 mg/kg IM) was given at 3–4 h post-surgery and then every 4–6 h during the first postoperative day. Meloxicam (1 mg/kg PO q24h) was initiated 24 h after the initial pre-operative dose and continued for 3 days. Postoperative pain assessments included monitoring for bruxism, lethargy, prolonged recumbency, tachycardia, and anorexia, with additional analgesics administered as required.

### 2.5 Surgical techniques

#### 2.5.1 Anatomical considerations in sheep

The lateral abdominal wall is composed of the skin, subcutaneous tissue (including the cutaneous trunci), and three muscle layers: the external abdominal oblique, internal abdominal oblique, and transversus abdominis. Midway along the lateral flank, the aponeuroses of the external and internal oblique muscles contribute to the external rectus sheath, while the transversus abdominis forms the internal rectus sheath (29). These sheaths envelop the rectus abdominis muscle, which courses longitudinally along the ventral abdomen. Ventrally, their aponeuroses converge at the midline to form the linea alba, a fibrous structure commonly used for abdominal access due to its relative avascularity (30).

A caudal ventral midline laparotomy was performed for surgeries one and three (Figure 4). A skin incision was made along the linea alba, beginning approximately 2–3 cm caudal to the umbilicus and extending 15–20 cm caudally. Deviation into the rectus sheath or rectus abdominis may compromise the integrity of the closure (31). Next, the subcutaneous tissue was carefully separated using blunt dissection with Metzenbaum scissors and electrocautery to expose the linea alba. The linea alba was tented with tissue forceps, and a small stab incision was made to enter the abdominal cavity. Hemostasis was achieved as needed using electrocautery or ligatures. The linea alba and peritoneum were then incised layer by layer under digital guidance, with the surgeon's fingers used to shield underlying structures such as the omentum and intestines during entry (32, 33).

A left lateral flank approach (Figure 5) was used for surgery two following the development of acute ventral midline dehiscence in the first two of the eight ewes. This approach was selected due to its reliability and that it is commonly used for right-sided abdominal exploration or left-sided cesarean section in sheep (33). The ewes were positioned in right lateral recumbency and elevated approximately 30 degrees toward the surgeon using a cushion or rolled towels. The paralumbar fossa was aseptically prepared and draped. A 15–25 cm vertical or slightly oblique skin incision was made, starting approximately 5 cm ventral to the lumbar transverse processes and centered within the fossa. The incision was continued through the external abdominal oblique, internal abdominal oblique, and transversus abdominis muscles, either sharply or by blunt dissection along the muscle fibers in a grid approach. The peritoneum was incised in line with the underlying muscle.

Care was taken to avoid injury to the underlying viscera upon entering the peritoneal cavity. In term-gestation sheep, the abdominal wall is thin, and the rumen is often distended and could be located immediately beneath the incision (32, 33). In this position, the mid-pregnant multiparous uterus is highly mobile and may have moved to the right side of the abdomen. Finding the uterus body in the pelvic inlet and guiding yourself along the body to the first horn may be needed.

#### 2.5.2 CDH creation

At embryonic day 80 (E80; term ∼E140), ewes carrying two fetuses, previously confirmed on ultrasound, underwent midline laparotomy and the gravid, bicornuate uterus was gently externalized. To mimic the hallmarks of CDH hypoplastic lungs, herniation of the abdominal organs must occur early in pregnancy to allow interference with normal lung development through compression. Specifically, we selected E80 as it corresponds to an early stage of lung organogenesis, namely the pseudoglandular phase, as reported previously by our group (22, 34). Attempts to create diaphragmatic defects at earlier stages have been associated with a high rate of fetal demise (18). The uterus was carefully palpated, and the number of fetuses was confirmed (usually one per uterine horn). The side of the experimental fetus was determined, and the opposite horn was returned to the abdomen. The fetus was externally manipulated to ensure that it was in right lateral decubitus position, with the left chest underneath the anterior uterine wall. Two 4-0 silk stay sutures (Ethicon Inc, Somerville, NJ) were placed through the uterus and into the fetus’ chest wall to maintain fetal position. A small hysterotomy was made over the lower left chest. A left fetal thoracotomy exposed the left lung and diaphragm. A cotton tip applicator gently retracted the fetal lung cranially, and the central white fibres of the diaphragm were identified. A 23G needle tip punctured the diaphragm while fine scissors and small mosquito forceps enlarged the defect to a diameter of 1–1.5 cm. At least one of the four compartments of the ruminant stomach were carefully pulled up into the left chest. The fetal chest was closed with an interrupted 4-0 silk suture to re-approximate the fetal ribs, and a running 4-0 silk suture was used to close the fetal muscle and skin layers. Warmed normal saline (at least half of the estimated volume of lost amniotic fluid) along with Cefazolin (500 mg; Fresenius Kabi Canada, Toronto, ON, Canada) 500 mg and Gentamicin (150 mg; Gentocin 100 mg/mL; Intervet Canada Corp., Kirkland, QC, Canada) were infused into the amniotic cavity prior to uterine closure. The uterus was closed with a running #1 Vicryl suture (Ethicon Inc, Somerville, NJ).

The linea alba was closed with a simple continuous pattern using #1 Vicryl, with 1-0 PDS (Ethicon Inc, Somerville, NJ) interrupted sutures after every third Vicryl suture, except in the first ewes reinforced with horizontal mattress sutures of the same material. Subcutaneous tissue was approximated with a running 2-0 Monocryl (Ethicon Inc, Somerville, NJ), and skin closure was completed with a 2-0 subcuticular Monocryl suture (35). The incision was sprayed with Opsite Spray (Smith & Nephew Inc., Mississauga, ON, Canada), then sterile gauze was placed over the surgical site, followed by 3M™ Ioban™ 2 Antimicrobial Incise Drape (3M Canada, London, ON, Canada).

**Surgical Diaphragmatic Defect Creation (E80)—Step by Step**

**Figure 6:**
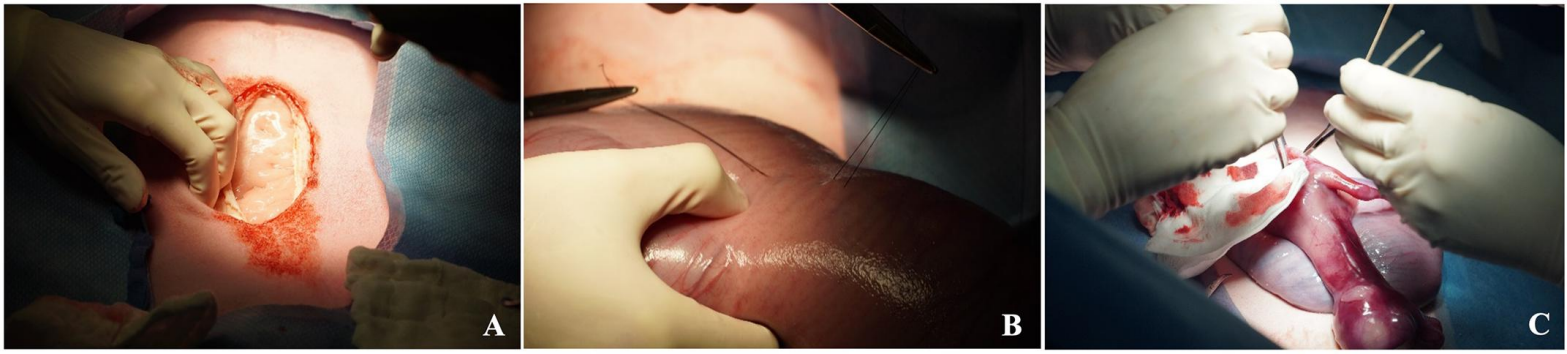
Maternal midline laparotomy approach **(A)**, exposure of the uterus and identification of the left fetal hemithorax. Stay sutures are then placed through the uterus into the fetal chest wall to maintain fetal position **(B)** It is recommended not to exteriorize the fetus as shown **(C)** but rather perform an intrauterine diaphragmatic hernia creation.

**Figure 7:**
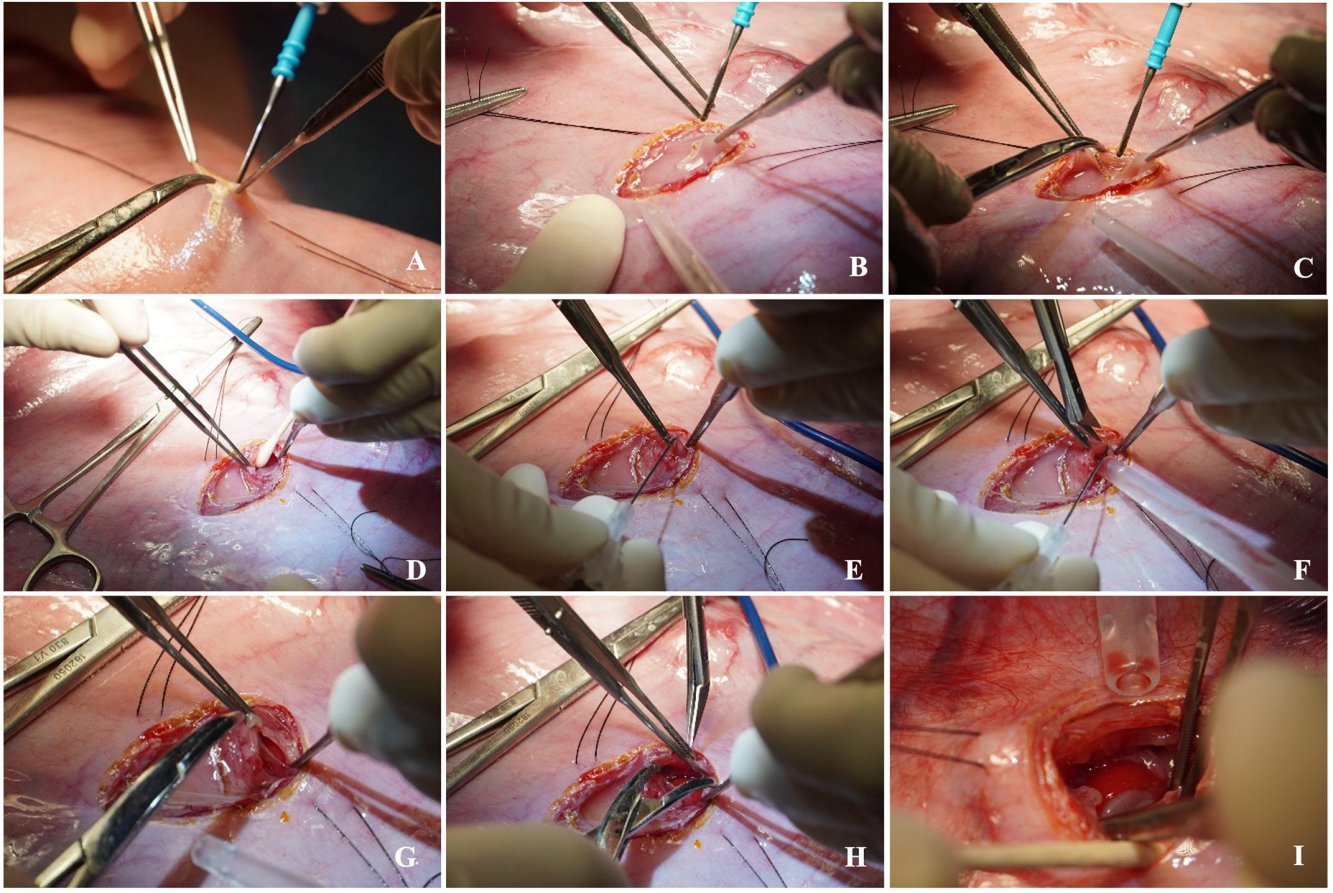
Step-by-step left fetal thoracotomy and hernia creation. A small hysterotomy is performed **(A)**, then the fetal lung and diaphragm are carefully exposed via left thoracotomy **(B–D)**. A diaphragmatic defect is created **(C)**, enlarged **(D–H)**, and a hernia is created by translocation of the abomasum into the thoracic cavity **(I)**.

**Figure 8:**
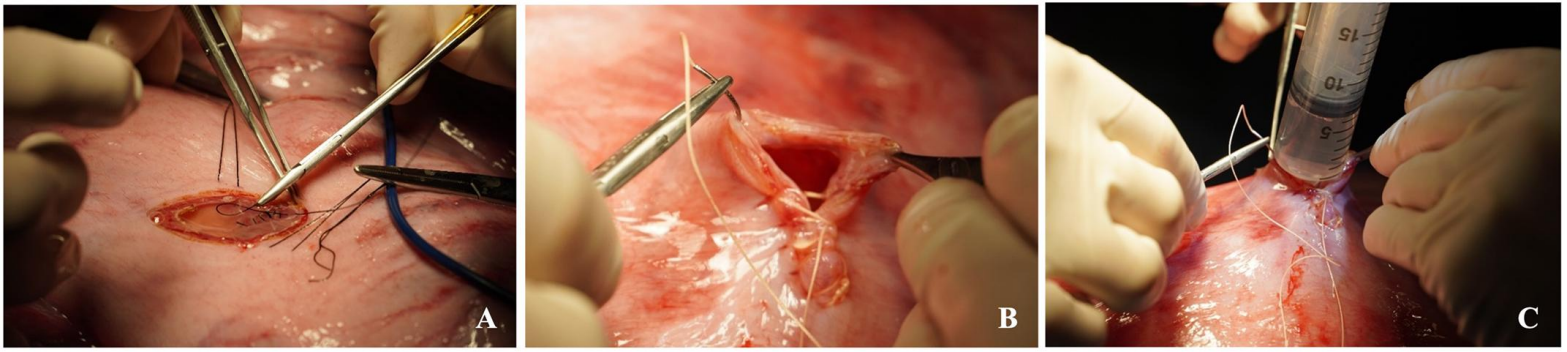
Closure of the fetal chest **(A)**, closure of the hysterotomy **(B)** and intra-amniotic antibiotic administration **(C)**.

**Figure 9:**
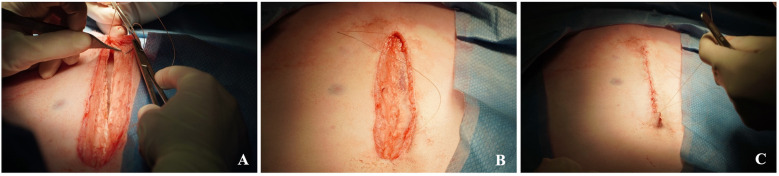
Closure of the maternal laparotomy. Re-approximation of the linea alba **(A)** and the subcutaneous tissue **(B)**, as well as the skin closure **(C)**.

1. ***Midline laparotomy***
    a. Skin incision via #10 blade (approx. 10–15 cm) (Figure 6A).
    b. Incision of the Linea alba and peritoneum via electrocautery.
  1. ***Exposure of the uterus and identifying the lamb fetus***
    a. Whenever possible, only a single uterine horn should be exteriorized to minimize uterine manipulation and reduce the risk of preterm labour.
  2. **Caveat:** Each uterine horn contains a substantial volume of amniotic fluid (approximately 700–1,000 mL), while the fetus occupies a relatively small proportion of the horn. The placenta is attached to the uterine wall by approximately 80 small structures or “buttons”, known as cotyledons, which are palpable through the uterine wall. These can make it challenging to localize the fetus.
  3. Via palpation, identify the head and snout of the fetus and confirm the position of the fetus via palpation of the spine (Figure 6B).
  4. Identify the left forelimb and rib cage of the fetus.
  5. **Caveat:** At approximately E80, the fetal neck is relatively elongated, and the thoracic cavity is positioned more caudally than expected.
  6. Note, the left forelimb and head of the fetus were exteriorized in Figure 6C as shown; however, this approach carries a significant risk of preterm fetal loss. Accordingly, it is recommended to not exteriorize the fetus but rather to perform the following steps described below using stay sutures while maintaining the fetus intrauterine.

2. ***Fetal thoracotomy and hernia creation:***
  7. After identification of the left fetal hemithorax (especially the postero-lateral aspect of the ribs), 2× 4-0 silk stay sutures will be placed through the uterus and into the fetus’ chest wall to maintain fetal position, this step is crucial to further proceed and to avoid massive amniotic fluid leakage (Figure 6B).
  8. Gerald forceps are used to handle the uterine wall, a small hysterotomy incision (3–4 cm max) is made via electrocautery (Figure 7A).
  9. The electrocautery and a fine mosquito are used to enter the fetal chest and expose the fetal lung and diaphragm (Figures 7B,C).
  10. A cotton tip applicator gently retracts the fetal lung and identifies the central white fibers of the diaphragm (Figure 7D).
  11. Using a 23-gauge needle (Figure 7E), carefully puncture the fetal diaphragm, advancing only the needle tip in an upward direction to create a controlled entry point while minimizing tissue disruption.
    a. A small incision is made with a Pott's scissor (Figure 7F).
  12. Use a small mosquito forceps to enlarge the defect to approximately 1–1.5 cm (Caveat: at this point avoid liver bleeding by gentling inserting the tip of the forceps to enlarge the defect) (Figures 7G,H).
    b. Gently retract **at least** the abomasum (one of four ruminant stomach compartments), into the left thoracic cavity using non-toothed intestinal grasping forceps to simulate herniation through the diaphragmatic defect (Figure 7I).

3. ***Closure of the fetal chest, hysterotomy and laparotomy***
  13. The fetal thoracic wall should be closed using a running 4-0 silk suture to approximate both the muscle and skin layers (Figure 8A). It is recommended to shorten the length of the suture to prevent inadvertent snagging during closure. Due to the extreme fragility of the fetal body wall; comparable in consistency to wet tissue paper, sutures can easily cut through the tissue. Therefore, meticulous and gentle handling is essential throughout the procedure. Rib approximation may also be considered to support thoracic integrity.
  14. Close the hysterotomy by an inverted continuous, locking 2-0 Vicryl suture (Figure 8B).
  15. Intra-amniotic application of Cefazolin 500 mg, Gentamicin 150 mg and warm saline (at least half of the amniotic fluid loss) prior to the final closure of the hysterotomy (Figure 8C).
  16. Place the uterus back into the abdomen and re-approximate the linea alba with two continuous #1 Vicryl sutures (Figure 9A).
  17. Reapproximate the subcutaneous tissue with a running 2-0 Monocryl suture and close the skin via running subcuticular 2-0 Monocryl suture as well (Figures 9B,C).
  18. The incision was sprayed with Opsite Spray (Smith & Nephew Inc., Mississauga, ON, Canada). Sterile gauze was placed over the surgical site, followed by 3M™ Ioban™ 2 Antimicrobial Incise Drape (3M Canada, London, ON, Canada) until the ewes were recovered and standing. Then, the gauze was removed and replaced with an adhesive non-woven wound dressing (PRIMAPORE™ Post-Operative Dressing). It was then covered with cotton, and a supportive elastic abdominal bandage (Elastikon Elastic Tape, 4 inch).

#### 2.5.3 Fetal tracheal occlusion

At E108, a left flank laparotomy was performed to access and exteriorize the uterine horn containing the CDH fetus. The developmental timepoint E108 is translationally relevant, as it corresponds to the canalicular stage of lung development in humans (34). At this fetal stage (∼17–27 weeks in human gestation), the diagnosis of CDH can be established via ultrasonography, and fetal interventions to promote *in utero* lung development can be performed (3). The fetal snout was palpated, and a hysterotomy was created at a new site, allowing externalization of the fetal head. Gentle traction was placed on the head, and the fetal neck was exposed. A small incision was made on the mid trachea. Two 3-0 Vicryl ties were placed posteriorly behind the trachea and were used to lift the trachea gently. A small tracheotomy was performed and the CDH fetus received either saline or the experimental therapy at this point of the surgery. The trachea immediately distal to the tracheotomy incision was then tied off twice using the same Vicryl suture. Warmed normal saline (at least half of the estimated volume of lost amniotic fluid) along with Cefazolin (500 mg; Fresenius Kabi Canada, Toronto, ON, Canada) 500 mg and Gentamicin (150 mg; Gentocin 100 mg/mL; Intervet Canada Corp., Kirkland, QC, Canada) were infused into the amniotic cavity prior to uterine closure. Significantly less amniotic fluid was usually lost during this surgery since the fetuses were larger with less amniotic fluid at baseline. Due to the difficulty in determining which fetus had been previously operated on, the uterus was now closed with a simple continuous 1-0 PDS suture to allow identification at the time of Cesarean section.

Transverse and internal abdominal oblique muscles were closed together, and external abdominal oblique muscle was also closed in simple continuous fashion using #1 Vicryl sutures. The subcutaneous tissues with the truncus cutaneous muscle and skin were closed sequentially with running 2-0 Monocryl sutures (35). The incision was sprayed with Opsite Spray (Smith & Nephew Inc., Mississauga, ON, Canada), then sterile gauze was placed over the surgical site, followed by 3M™ Ioban™ 2 Antimicrobial Incise Drape (3M Canada, London, ON, Canada).

**Fetal Tracheal Occlusion Procedure—Step by Step**

Fetal tracheal occlusion was performed via a maternal left flank approach. This incision was separate from that used during the earlier CDH creation.

**Figure 10:**
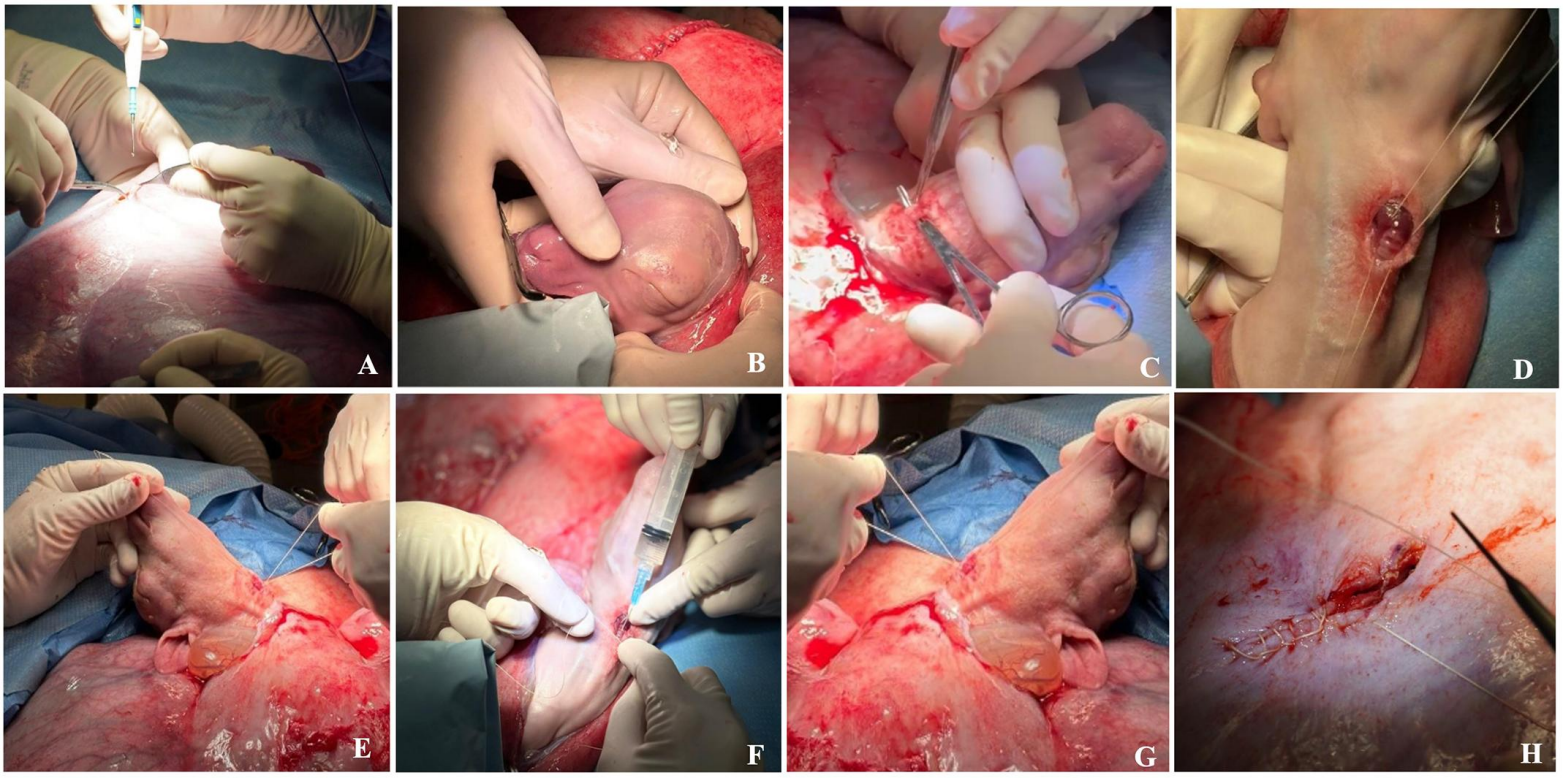
Step by step fetal tracheal occlusion procedure performed via a maternal left flank approach: **(A)** A small hysterotomy. **(B)** Delivery of the fetal head and neck. **(C)** Fetal median neck skin incision and trachea preparation/dissection with mosquito forceps. **(D)** Suture is placed below and above the tracheal injection site. **(E)** Proximal suture is tied; the knot of the distal suture is kept loose. **(F)** Therapeutic is injected into trachea. **(G)** Trachea is occluded. **(H)** Hysterotomy closure. Details are summarized in Section 2.5.3: Fetal Tracheal Occlusion Procedure—Step by Step.

1. ***Left flank approach***
  19. A 15–25 cm vertical or slightly oblique skin incision was made, starting approximately 5 cm ventral to the lumbar transverse processes, and centered within the fossa.
    a. The incision was continued through the external abdominal oblique, internal abdominal oblique, and transversus abdominis muscles, either sharply or by blunt dissection along the muscle fibers in a grid approach. The peritoneum was incised in line with the underlying muscle.
    b. Only one uterine horn was exteriorized at a time. Gerald forceps are used to gently handle the uterine wall. A small hysterotomy, approximately 5–6 cm in length, was created using electrocautery to minimize bleeding and tissue trauma (Figure 10A).

2. ***Fetal Tracheal Ligation and Occlusion***
    a. Delivery of the fetal head and neck (Figure 10B).
  20. Fetal median neck skin incision (approximately 2–3 cm) and gentle preparation with mosquito forceps towards the trachea (Figure 10C).
    a. Gentle preparation/dissection with mosquito forceps behind the trachea (Careful due to its proximity to the internal carotid vessels) (Figure 10C)
  21. 3-0 Vicryl is placed below and above the tracheal injection site (Figure 10D).
    a. Proximal suture is tied; the knot of the distal suture is prepared but kept loose (Figure 10E).
    b. Subsequently, the therapeutic treatment of choice is injected into the trachea via a 24G needle (Figure 10F).
    c. The suture is then tied down to occlude the trachea (Figure 10G).
  22. The fetal neck incision was closed with a running, locking 4-0 Vicryl suture.

3. ***Maternal Closure***
  23. Hysterotomy closure by an inverted continuous, locking suture via 2-0 Vicryl (Figure 10H).
    a. Intra-amniotic application of Cefazolin 500 mg, Gentamicin 150 mg and warm saline (at least half of the amniotic fluid loss) (Figure 8C).

#### 2.5.4 Cesarean section and delivery

At E136, ewes were euthanized prior to cesarian section with an intravenous administration of pentobarbital sodium (1 mL per 5 kg BW or 10 mL per 50 kg BW; Euthanyl Forte; 540 mg/mL; Bimeda-MTC Animal Health Inc, Cambridge, ON) and lambs were delivered via midline laparotomy. Maternal administration allowed transplacental circulation of pentobarbital to the fetal lamb. Delivery was scheduled at this pre-term stage to permit assessment of treatment induced differences before the onset of postnatal oxygenation, which would have altered pulmonary architecture. If a fetal heartbeat was detected after hysterotomy, an umbilical cord injection of pentobarbital sodium was performed, followed by an intracardiac injection if necessary. The umbilical cord was then clamped, and the lamb delivered.

#### 2.5.5 Important veterinary and technical considerations

Sheep are highly social animals with strong flocking behaviour that reduces individual stress response when handled in groups (26). To accommodate this, sedation of each ewe was staggered at one-hour intervals to avoid repeated or unnecessary stress. Care was taken during both the preoperative and postoperative periods to ensure that each ewe maintained physical or visual contact with conspecifics, as the surgical procedure required approximately 60 min. At all times, at least three sheep were penned together in one large pen.

Following surgery, ewes were recovered in a padded large animal induction/recovery stall. Once the ewes stood up, the previously applied 3M™ Ioban™ 2 Antimicrobial Incise Drape (3M Canada, London, ON, Canada) was removed. The incision was then dressed immediately with an adhesive non-woven wound dressing (PRIMAPORE™ Post-Operative Dressing). It was then covered with cotton and a supportive elastic abdominal bandage (Elastikon Elastic Tape, 4 inches).

**Figure 11:**
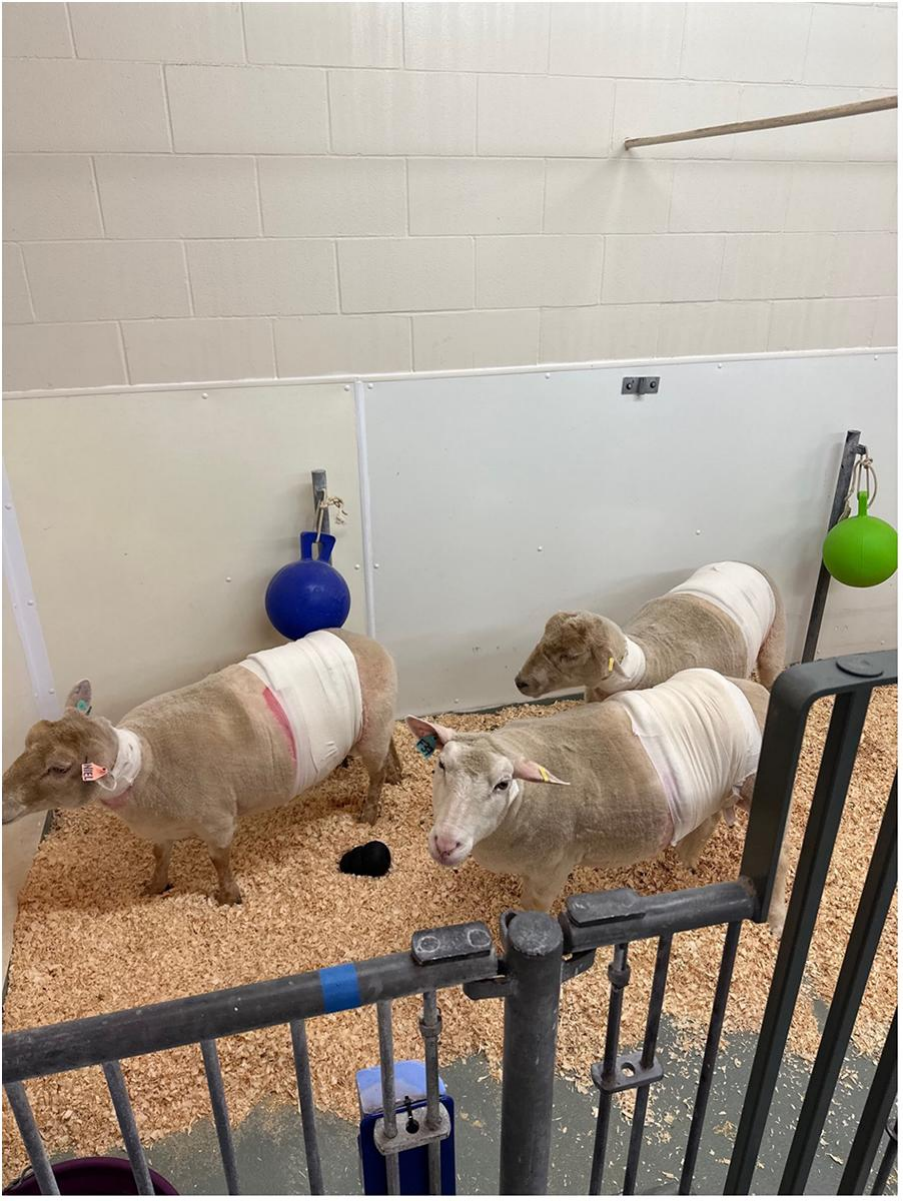
Postoperative housing for ewes following abdominal surgery. Ewes were group-housed in a climate-controlled facility. Pens were bedded with clean wood shavings and contained environmental enrichment. Feed, water, and enrichment were provided *ad libitum*, and animals were monitored closely by veterinary and animal care technicians.

The ewes were recovered in individually partitioned pens within the larger pen and sight of the other sheep. Once consistently standing and clinically stable, they were returned to the larger pen (Figure 11). Food and water were reintroduced following ruminant refeeding protocols of the Ontario Veterinary College Large Animal Hospital. Postoperatively, the ewes were closely monitored by veterinarians at two-hour intervals for the remainder of the day until 11:00 pm and then checked every twelve hours for two days after surgery, and then once a day thereafter until the following surgeries and subsequent euthanasia procedures.

The clinical examination included HR, RR, ruminal contraction rate, rectal temperature, mucous membrane colour and capillary refill time. The surgical site was assessed daily by visual inspection and gentle palpation to detect swelling, redness, pain, abnormal discharge, or signs of surgical site infection (SSI) or incisional herniation. Concurrent behavioural monitoring observed for signs of pain or discomfort such as teeth grinding, reluctance to rise, prolonged recumbency, pawing, licking the incision site, or social withdrawal. Additional parameters included body weight, BCS, appetite, water intake, activity level, fecal output, and urination, if observed. It is assumed that minimal restraint was required during handling due to the thorough preoperative acclimation period with animal care staff and veterinarians. As previously mentioned, all observations were documented on the SOAP and day-to-day assessment point system post-surgery records (Figures 2, 3).

**Figure 12:**
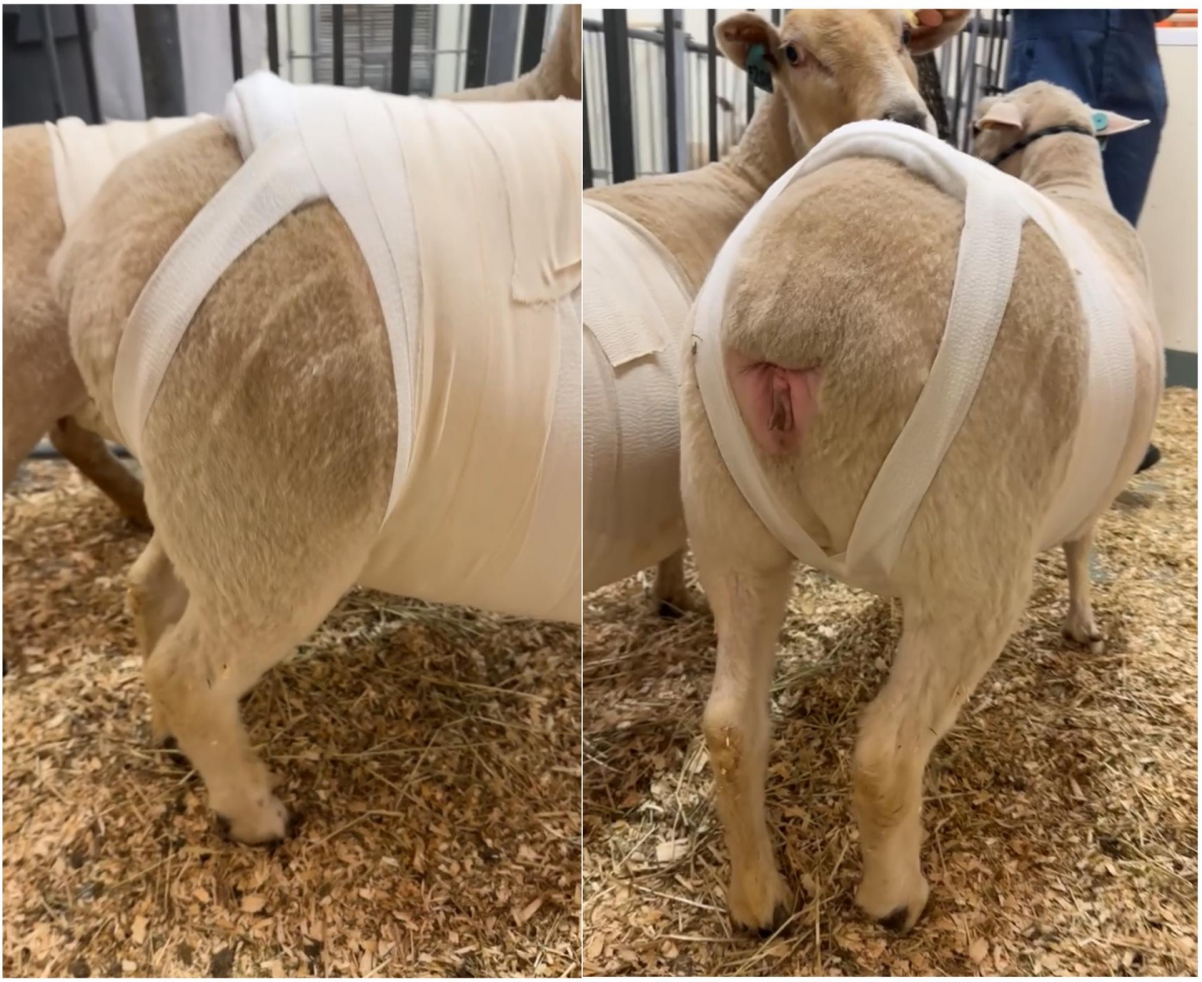
Application of a supportive “truss” abdominal bandage (elastikon elastic tape, 4 inch) following abdominal surgery in ewes. The bandage was initiated over the sternum and applied caudally with overlapping layers to the flank, carefully excluding the udder. The cross-strapping “truss” technique provided uniform pressure over the ventral abdominal incision to reduce the risk of herniation or further tissue protrusion, while maintaining comfort and mobility during recovery.

Incisional herniation occurred in two of eight ewes, resulting in complete midline dehiscence within six days post-surgery, with one ewe progressing to evisceration. Ultrasound with a convex array transducer (Philips Lumify, C5-2; 2–5 MHz) was used to assess suspected hernias. Following the presentation of hernia in these ewes, it was elected to apply a truss bandage using sheet cotton, and a supportive elastic abdominal bandage (Elastikon Elastic Tape, 4 inch). The bandage was initiated over the sternum, applied caudally with overlapping layers up to the last rib at the flank area, and carefully excluded the udder (Figure 12). The cross-strapping “truss” technique provided uniform pressure over the ventral abdominal incision, helping reduce the risk of herniation or tissue protrusion while maintaining ewe comfort and mobility. For larger hernias, additional rolled cotton is recommended to increase localized pressure and aid in reduction. Care must be taken to avoid obstructing the anus or prepuce with the truss bandage. Therefore, the bandage should be checked several times daily to ensure proper placement and to monitor for any signs of skin irritation, allowing for timely adjustment or reapplication if needed.

In the ewe with evisceration, humane euthanasia was performed using IV pentobarbital sodium, preceded by sedation with an alpha-2 agonist. In the second ewe, significant abdominal wall damage precluded surgical repair, and euthanasia was elected to avoid the risk of evisceration. As previously discussed, these complications prompted several protocol refinements; 1) ensuring that the midline laparotomy began closer to the umbilicus and did not extend too far caudally, 2) additional interrupted sutures during midline closure, and 3) switching the surgical approach from the ventral midline to the left flank approach for the second surgery.

Acute hernias within seven days post-op carry a greater evisceration risk, as skin strength has not yet recovered (36, 37). Sheep have a thinner body wall and more retroperitoneal fat than cattle or goats, predisposing them to herniation (38). Other contributing factors include poor suture technique (36), infection (36, 39, 40), impaired healing, increased intra-abdominal pressure (36), prolonged surgery (40), and postoperative activity (39). When palpation is inconclusive, ultrasound confirms diagnosis and guides repair planning (39).

## 3 Discussion

Sheep are a well-established large animal model for prenatal surgical studies, particularly in the context of lung development and research surrounding CDH. Their anatomical and physiological characteristics, including symmetrical airway branching, the presence of cartilage in the upper airway, and the onset of alveolar development near term, closely parallel those of human fetuses (41). In addition, their larger organ size enables diagnostic imaging, surgical approaches, and post-mortem evaluations similar to humans, making them particularly well suited for translational applications.

This methodology paper outlines the refinement of a fetal sheep model of CDH with a focus on veterinary considerations, surgical approach, and animal welfare. Although FETO has emerged as a promising prenatal therapy in both clinical and experimental settings, it was not performed in the present study as this initial phase was dedicated to refining the model with a particular focus on animal welfare and veterinary optimization, and the required fetoscopic equipment was not yet available at our institution. Due to this, tracheal ligation was used to achieve airway occlusion through a technically simpler open approach. In the next phase of our work, we will use this refined model in combination with FETO to more closely replicate the current clinical standards in human CDH, as well as to minimize maternal complications associated with open approaches. Importantly, by adapting the model to FETO, it will also permit us to investigate the targeted delivery of a novel therapeutic, advancing the translational applicability of our work to human CDH patients.

One of the key improvements in our surgical approach was prompted by incisional herniation observed in the initial cases using a ventral midline laparotomy. While this initial approach offered excellent uterine access and is preferred when both horns must be visualized (42, 43), it carries a risk of incisional complications, especially in late gestation and twin pregnancies. Subsequently, this first approach was determined to be too caudal and led to complications, including incisional herniation and evisceration. The complications may have been worsened by the sheep's thin abdominal musculature (43), along with the added strain of carrying twin (*n* = 7) or triplet (*n* = 1) pregnancies. Notably, most surgical protocols in the literature are based on singleton pregnancies. In response, we initiated the additional ventral midline approaches at the umbilicus, with less caudal extension. The second laparotomy was performed through a left flank approach to minimize incisional complications. Although the left flank approach was associated with slightly more postoperative discomfort in the ewes, it avoided herniation entirely. In this regard, this approach benefits from the rumen's supportive positioning, which has been postulated to reduce intra-abdominal pressure-related complications (43). In the ewes presenting with increased pain, this was addressed with appropriate analgesia and postoperative care by the veterinary team.

While the primary aim of this initial work was surgical refinement rather than comparative evaluation between various approaches, we acknowledge that the lack of formal quantitative scoring of maternal recovery and fetal viability between approaches is a limitation. Qualitatively, however, the divergence in clinical outcomes was clear; the initial ventral midline surgeries resulted in postoperative deterioration requiring euthanasia, whereas all ewes that underwent the left flank approach recovered uneventfully with no fetal losses prior to the planned terminal procedure. Future work by our group, and others, should incorporate quantitative or semi-quantitative maternal pain and recovery, as well as fetal monitoring metrics, to enable comparisons between surgical techniques and continued refinement of the model for improved animal welfare and better translation to human patients.

Our prophylactic use of a “truss-style” abdominal bandage was also introduced as a refinement in our approach to support the ventral incision. While it's unclear whether bandaging, surgical technique adjustments following herniation, or both, were primarily responsible for improved outcomes, the combination likely minimized early wound stress during the critical healing window, especially given the recumbency patterns of sheep compared to species like horses which our veterinary team commonly treats in hospital. Protecting the surgical site in the initial 24–72 h, combined with bandaging and monitoring for swelling or discharge, aligned with recommendations for preventing surgical site infections in large animals (44–46).

Preoperative management was adapted to reduce the risk of aspiration and regurgitation, with moderate feed and water withdrawal consistent with ruminant anesthesia guidelines (47, 48). Excessive fasting, known to disrupt rumen motility and microbial balance (26, 48), should be avoided, and may be supported by supplementing with intravenous fluids (49). Risk of active or passive regurgitation was minimized by performing induction in sternal recumbency, elevating the head, and using rapid intubation with a cuffed endotracheal tube (47, 49). Anesthetic protocols incorporated low-dose xylazine, as sheep are highly sensitive to alpha-2 agonists and may develop hypoxemia or pulmonary edema if overdosed (50).

Pain management strategies focused on multimodal analgesia. Long-acting local anesthetics such as bupivacaine were used intraoperatively, while postoperative regimens included hydromorphone and meloxicam. The latter was selected for its good oral bioavailability and effective dosing in sheep (51). Animal welfare occupied a significant portion of this study, and the ewes were routinely assessed for pain and distress. Pain and distress were evaluated through vitals (e.g., heart and respiratory rate) and behavioural assessments, which included monitoring for signs such as bruxism, ear droop, squinting, prolonged recumbency, reluctance to rise, social withdrawal or incision licking. Additional indicators included appetite, fecal output, and activity level. While not a formal pain scale, they reflect established clinical welfare practices (26, 27). Recent developments in composite and facial expression-based pain scoring systems support integrating these observations into postoperative monitoring in sheep (52–54).

Tocolytics were not administered in our study as all ewes received meloxicam post-operatively. Meloxicam has effectively inhibited preterm labour-like uterine contractions in sheep (55), which may reduce the need for additional tocolytics like medroxyprogesterone acetate. Moreover, progesterone supplementation beyond embryo implantation has not been shown to improve pregnancy outcomes in ewes and is not standard practice (56); therefore, we did not administer it in our study.

**Figure 13:**
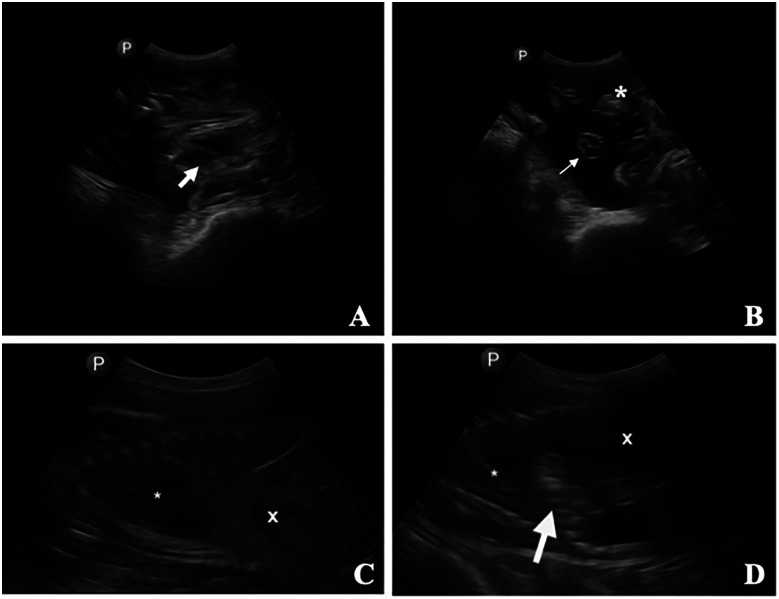
Transabdominal ultrasonographic images of a pregnant ewe. **(A)** Fetal heart (thick arrow). **(B)** Umbilical cord (thin arrow) and placentomes (*). **(C)** Fetus in one ewe, with the stomach (*) and liver (X) identified. **(D)** Second fetus in the same ewe, with the stomach (*), liver (X), and a small gas-filled section of the rumen (arrow) displaced into the thorax between the heart and the liver/diaphragm (demonstrating the surgically created diaphragmic hernia).

Transabdominal ultrasound was used to confirm fetal heartbeat (Figures 13A,B), maternal integrity of the uterine and abdominal walls, and subsequent fetal surgical CDH creation (Figures 13C,D). This technique also proved useful in detecting early signs of incisional herniation prior to clinical deterioration, which meant that we could execute timely interventions or euthanasia if deemed necessary.

Finally, the CDH model benefited from careful attention to species-specific behavioural needs, reinforcing the critical role of veterinary oversight in animal research. In this study, housing strategies minimized social isolation, and the prolonged preoperative acclimation period we utilized allowed for baseline behaviour assessments and, most importantly, stress reduction. Positive reinforcement during routine handling helped promote a positive response from the ewes during daily assessments and postoperative care.

In conclusion, this refined fetal sheep model of CDH balances surgical approach, maternal recovery, and animal welfare to provide a reliable and ethically sound platform for prenatal translational research. This model offers a reproducible and clinically relevant approach to evaluating fetal therapies for CDH through improvements to anesthesia and analgesia, surgical technique, and postoperative care protocols. In this methodology paper, we especially emphasize the critical role of a cross-disciplinary team of pediatric surgeons, scientists, and veterinary specialists, whose combined expertise is essential to advancing translational work while upholding the highest standards of animal care and welfare.

## Data Availability

The original contributions presented in the study are included in the article/Supplementary Material, further inquiries can be directed to the corresponding author/s.
