# Supplementary material for: Veterinary and technical optimization of the fetal sheep model of congenital diaphragmatic hernia: implications for translational pediatric surgery
Source: Front Surg. 2025 Dec 3;12:1711870. doi: 10.3389/fsurg.2025.1711870 (PMC12708541; doi:10.3389/fsurg.2025.1711870)
Supplement: Supplementary file 1 [file Datasheet1.pdf]

Supplementary Material

Surgical Supplies

**Material**	**Size**	**Amount**
Sterile surgical gloves	As applicable	1
Sterile surgical gown	As applicable	1
Surgical prep sponge (chlorhexidine gluconate 2%)	n/a	1
Vicryl	1	2
Vicryl	2-0	1
Monocryl	2-0	2
Silk	4-0	1
Poole Surgical Suction Instrument	Preferable as small as possible	1
Connecting tube	1/4in x 120in	
Light handle cover	n/a	2
Table cover	44in x 90in	
Laparotomy drape	77in x 122in x 106in	1
Syringe	20cc	1
Syringe (catheter tip)	50cc	1
Needle	23G	2
Blade	#10	1
Cotton tip applicator	3in	2-3
Sterile Saline	500cc	1
Electrosurgical cautery device	n/a	1
Cautery blade/pencil tip	2¾ in	1
Sterile gauze		5
Sterile laparotomy sponges	12 in x 12 in	5
Antibiotics	Cefazolin 500mg<br>Gentamycin 150mg	2
**Surgical Tray Components**	**Size**	**Amount**
Surgical cup	2 in	
Surgical bowl	4 in	1
Scalpel handle	5 in	1
Needle driver	6¼ in	1
Derf needle holder (TC insert)	4½ in	1
Forceps hemostat (straight)	5½ in	2
Delicate mosquito forceps (curved)	5 in	2
Adson tissue forceps (1x2 teeth)	4¾ in	2
Gerald tissue forceps serrated	7 in	2
Stevens scissor curved	4½ in	1
Suture scissor	5¾ in	1
Diethrich-Pott's vascular scissors (45 degree)	5⅜ in	1
Singley tissue forceps	9 in	1
